# Factors Influencing Public Attitudes and Willingness to Utilize Telepharmacy Services in the UAE

**DOI:** 10.1155/2024/5755493

**Published:** 2024-08-19

**Authors:** Anan S. Jarab, Walid Al-Qerem, Tareq Mukattash, Ahmad Al-Azayzih, Zelal Kharaba, Shrouq Abu Heshmeh, Joud Al-Momani, Rama Hamdan, Yazid N. Al Hamarneh, Judith Eberhardt

**Affiliations:** ^1^ College of Pharmacy AL Ain University, PO Box 122612, Abu Dhabi, UAE; ^2^ Department of Clinical Pharmacy Faculty of Pharmacy Jordan University of Science and Technology, PO Box 3030, Irbid 22110, Jordan; ^3^ Department of Pharmacy Faculty of Pharmacy Al-Zaytoonah University of Jordan, PO Box 130, Amman 11733, Jordan; ^4^ Department of Pharmacy Practice and Pharmacotherapeutics College of Pharmacy University of Sharjah, Sharjah, UAE; ^5^ Department of Pharmacology Faculty of Medicine and Dentistry University of Alberta, Edmonton, Canada; ^6^ Department of Psychology School of Social Sciences Humanities and Law Teesside University, Middlesbrough, UK

**Keywords:** attitude, community pharmacy services, online, patient acceptance of healthcare, pharmaceutical services, public opinion

## Abstract

**Background:** Telepharmacy, utilizing telecommunications to dispense pharmaceutical products and deliver patient care, offers numerous benefits for both the public and pharmacists. Previous research on exploring attitudes and willingness to use telepharmacy services has primarily focused on pharmacists rather than the general population.

**Aim:** This study is aimed at assessing the attitudes and willingness of the United Arab Emirates (UAE) population to utilize telepharmacy services and identifying the factors influencing their inclination to use these services.

**Methods:** In this cross-sectional study, a survey was distributed using convenience and snowball sampling to individuals aged 18 or older across the UAE through various social media platforms, including Twitter, Facebook, and WhatsApp. The survey domains included sociodemographics, attitudes, and readiness to utilize a telepharmacy service. A binary logistic regression analysis was conducted to investigate the variables associated with participants' willingness to utilize telepharmacy in the future.

**Results:** In total, 963 individuals participated in the study. Participants showed overall positive attitudes towards telepharmacy, with 70.9% believing that telepharmacy saved time and effort. While only 32% of the participants acknowledged that numerous telepharmacy services were available for use in the UAE, most were interested in using telepharmacy services in the future (79.2%). Participants who had higher attitude scores (AOR = 1.147, 95% confidence interval [CI]: 1.11–1.18) and those who had used these services previously (AOR = 3.270, 95% CI: 1.692–6.320) were more interested in using telepharmacy services in the future.

**Conclusion:** Forthcoming healthcare strategies should focus on expanding the availability of telepharmacy services throughout various regions of the country. This expansion will facilitate the broader utilization of these services and ultimately contribute to improved health outcomes.

## 1. Introduction

Telemedicine involves the remote delivery of medical services and healthcare via the use of information technology and communication tools [1]. Among the varied services offered is telepharmacy, a method for dispensing pharmaceutical products and providing care to patients using telecommunications. This innovative approach allows patients to conveniently receive medications and other pharmaceutical care services in the comfort of their surroundings and includes providing patient counselling, monitoring drug therapy, allowing prescription refills, and ensuring medication compliance [2]. Telepharmacy has the potential to revolutionize the way healthcare services are delivered, particularly in areas where access to healthcare services can be challenging, such as rural areas and underserved communities. Notably, there is internet access in rural and distant regions of the United Arab Emirates (UAE). In February 2024, the UAE had 9.46 million internet users, and the internet penetration rate stood at 99% of the total population. Given that the population was 9.55 million in January 2024, this indicates widespread internet access. While 87.9% of the UAE's population lives in urban centers, around 12.1% reside in rural areas, which also have internet access [3].

While telepharmacy is increasingly recognized globally for its potential to improve healthcare access and efficiency, there is a notable gap in the literature concerning its acceptance and implementation within the specific socioeconomic and cultural context of the Middle East, particularly in the UAE. This region presents unique challenges and opportunities due to its diverse population, rapid technological advancement, and distinctive healthcare system dynamics. The rapid growth of the UAE's population has significantly influenced its healthcare system, necessitating an increase in healthcare facilities and workers to meet this demand. Furthermore, the UAE's approach to healthcare is heavily influenced by its commitment to becoming a regional hub for medical tourism, which aligns with its strategic goals to enhance service quality and healthcare standards [4].

The utilization of telepharmacy has yielded several advantages for both patients and pharmacists. It has been found that telepharmacy effectively reduces travel expenses and time consumption, addressing crucial concerns, particularly for individuals in remote and rural areas seeking healthcare services, including disabled and elderly populations [5, 6]. In addition, telepharmacy has enhanced the clinical responsibilities of pharmacists, affording them ample time for confidential drug counselling, and thereby increasing the effectiveness of their clinical roles [7, 8]. Furthermore, prior research has shown a reduction in medication errors following the implementation of a telepharmacy service [9]. Despite these global advancements, the adoption of telepharmacy in the UAE remains underexplored, especially regarding the public's attitudes and willingness to utilize these services. Understanding these aspects is important, as the successful implementation of health innovations often hinges on public acceptance. The focus on the UAE is particularly relevant given the push towards digitalizing its healthcare services as part of its Vision 2021 strategy, which prioritizes smart health technologies [10].

Studies conducted in various countries have shown promising findings regarding the public acceptance of telepharmacy services [11–14]. The success of telepharmacy services depends not only on the advancement of technology and regulatory frameworks but also on the attitudes and willingness of people to adopt and use these services. Telepharmacy is an essential component of the broader concept of integrated pharmacy, enabling remote pharmaceutical services to be seamlessly integrated into comprehensive healthcare systems that demonstrate a beneficial impact on primary healthcare [15]. For the successful implementation and integration of telepharmacy services into the healthcare system, it is crucial for policymakers, healthcare professionals, and other stakeholders to understand consumer attitudes towards and willingness to use these services. Therefore, the current study sought to examine the attitude and willingness of the public in the UAE towards telepharmacy services, in addition to exploring the factors associated with their interest in using these services in the future. The results of this study were expected to offer valuable insights for devising effective strategies to broaden the scope of telepharmacy applications in the UAE.

## 2. Materials and Methods

### 2.1. Study Design and Participants

In this cross-sectional study, a self-administered online survey was distributed to the public in the UAE using convenience and snowball sampling between February and August 2023. Residents of the UAE aged 18 years or older were eligible to participate. A research assistant distributed the survey through several social media platforms, including Twitter, Facebook, and WhatsApp, and encouraged potential participants to invite others from their network to participate. The survey started with an introduction outlining the aims of the study and emphasizing participants' anonymity.

### 2.2. Data Collection Tool

The study survey was developed from a review of relevant literature [8, 10]. The data collection tool used in the current study underwent face and content validation through an expert panel review, including a professor in public health, a professor in pharmacy practice, and three community pharmacists. Next, the survey was piloted with 10 individuals to assess its clarity and relevance. Data from the pilot study was excluded from the main study findings. The survey was structured into three sections. The first section gathered sociodemographic information from participants and inquired about their prior experiences with telepharmacy and their interest in using it in the future. The second section consisted of nine items that identified the most common pharmaceutical products sought after by participants from community pharmacies. The final section included seven items designed to assess participants' attitudes towards telepharmacy. These items were rated on a 5-point Likert scale, ranging from *strongly disagree* to *strongly agree*. Subsequently, the scores from this section were totaled to generate an overall attitude score.

### 2.3. Statistical Analysis

**Figure 1:**
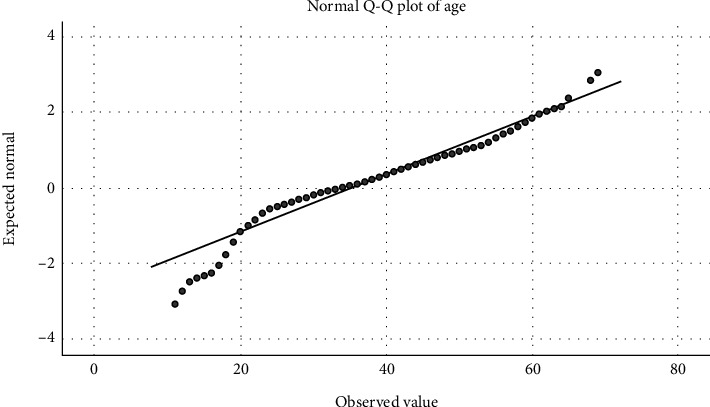
Q-Q plot for age.

**Figure 2:**
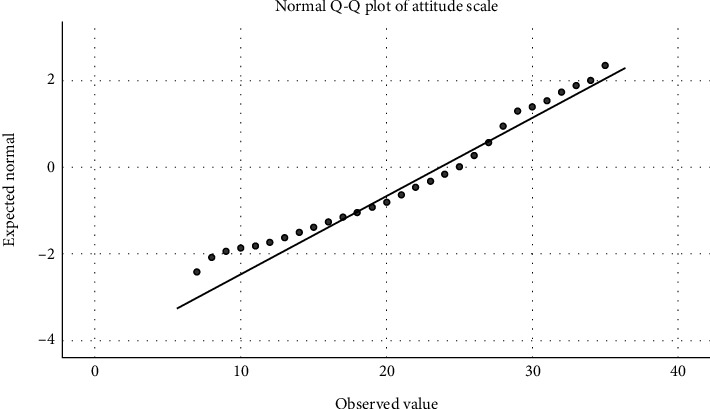
Q-Q plot for attitude scale.

Statistical analyses were conducted using the Statistical Package for the Social Sciences (SPSS, Version 28). Q-Q plots (Figures 1 and 2) and a Kolmogorov–Smirnov test with *p* < 0.001 revealed that continuous variables were not normally distributed and therefore were presented as medians and 95% confidence intervals (CIs). On the other hand, categorical variables were presented as frequencies (percentages). A binary logistic regression analysis was conducted to explore the variables associated with the dependent variable, which was participants' interest in using telepharmacy in the future, as assessed with the question “Are you interested in using telepharmacy services in the future?” The independent variables included age, sex, marital status, education, working status, income, insurance coverage, difficulty reaching the pharmacy, chronic diseases, attitude scores towards telepharmacy, and previous use of telepharmacy services as independent variables. These variables were chosen based purely on theoretical aspects of possible contributors to the intention to use telepharmacy services in the future. Multicollinearity was evaluated by computing VIF and tolerance for each independent variable. Model fitness was assessed by conducting a likelihood-ratio test and producing a Nagelkerke *R*^2^ value. Significance was determined at *p* < 0.05.

### 2.4. Ethics Approval

The study was conducted in line with the principles of the Declaration of Helsinki. The current study received ethics approval from the research ethics committee at Al Ain University-Abu-Dhabi Campus (ref. no. COP/AREC/AD/08). Informed consent was obtained from participants prior to their participation in the study.

## 3. Results

In total, 963 participants were included in the present study. The mean age was 34 (ranging from 33 to 36) years. Most of the participants (74.2%) were female, had a bachelor's degree (62.4%), had a low monthly income (60.2%), were not working (78.1%), or had relatives working in the healthcare sector (62.5%). Only 15.2% of the participants self-reported that they had a chronic condition, of whom 88.3% were on chronic medication and 49.2% followed a specific diet. Around 45.2% of the participants had a family member who suffered from a chronic condition; of those, 96.2% and 45.8% were taking chronic medication and following a specific diet, respectively (Table 1).

**Figure 3:**
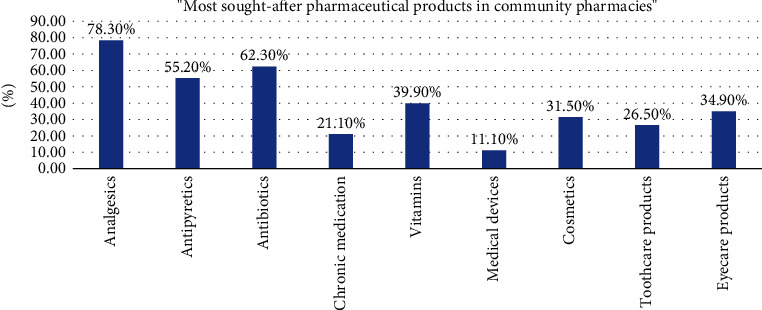
Most sought-after pharmaceutical products in community pharmacies (*n* = 963).

As shown in Figure 3, most participants used their community pharmacy to get analgesic drugs (78.3%), antibiotics (62.3%), and antipyretics (55.2%). The least sought-after pharmaceutical products were medical devices (11.1%) and chronic medications (21.1%).

Table 2 illustrates how participants responded to attitude-related items. The median attitude score was 25 (ranging from 25 to 26) out of a maximum possible score of 35. A significant majority, exceeding 70% of participants, expressed agreement or strong agreement with the idea that telepharmacy saved time and effort (70.9%). On the other hand, only 32% of participants agreed or strongly agreed that there were numerous telepharmacy services available for use in the UAE. Cronbach's alpha of the attitude scale was 0.88, indicating acceptable reliability. All items had a corrected item–total correlation above the cutoff point of 0.3. Cronbach's alpha would be reduced if any of the items were deleted, except for the item “I can use many telepharmacy services in the United Arab Emirates,” for which deleting it would increase Cronbach's alpha to 0.9. However, although removing this item would slightly increase the reliability by 0.02, it would affect the theoretical aspect of the scale. Given that the item's item–total correlation was acceptable (0.38), it was decided to retain the item.

A binary logistic regression analysis was conducted to explore the variables associated with participants' interest in using telepharmacy in the future, as evaluated by the question “Are you interested in using telepharmacy services in the future?” The results (see Table 3) showed that participants who had a higher attitude score (AOR = 1.147, 95% CI: 1.11–1.18) and those who had used telepharmacy services previously (AOR = 3.270, 95% CI: 1.692–6.320) had higher odds of answering “yes” to the question “Are you interested in using telepharmacy services in the future?” The VIF/tolerance for all the independent variables was within the acceptable range, confirming the lack of multicollinearity. The likelihood ratio test confirmed the model's fitness with *p* < 0.001. The Nagelkerke *R*^2^ value indicated that the model explained 17.1% of the outcome variance.

## 4. Discussion

The findings of the current study are useful for understanding, implementing, and promoting telepharmacy acceptance in the UAE. The findings showed a positive willingness and attitude towards telepharmacy. Having more favorable attitudes towards telepharmacy and prior use of this service were significantly and independently associated with greater interest in its implementation in the future.

Although most participants in the present study had not used telepharmacy services before, they expressed an interest in using them in the future. Research conducted in Jordan and Indonesia has indicated that the general population in these countries is open to utilizing telepharmacy services [13, 16]. The appeal of telepharmacy for participants may lie in its convenience, as it allows them to consult with pharmacists and obtain medications without leaving their homes, potentially saving time and effort compared to regular pharmacy visits. This high level of interest points to a potentially sizable market opportunity for telepharmacy services, which could promote their expansion and enhance access to healthcare services, especially in underserved or distant regions where access to physical pharmacies might be limited.

In the current study, the majority of participants visited community pharmacies primarily to obtain analgesic drugs, antibiotics, and antipyretics. This finding aligns with research conducted in Pakistan, where most participants also sought analgesics and antipyretics at community pharmacies [17]. Similarly, a study conducted in Ethiopia revealed that analgesics were the most commonly utilized drugs by participants visiting community pharmacies [18]. In light of these findings, knowing the community's particular medication preferences enables telepharmacies to customize their services better to handle the most common health issues efficiently.

Overall, participants in the current study exhibited favorable attitudes towards telepharmacy, which was in line with findings reported in earlier studies conducted in Malaysia [19], Saudi Arabia [11, 12], Indonesia [13], and Vietnam [14]. In the present study, while nearly three-quarters of participants believed that telepharmacy saved time and effort, only around a third of participants acknowledged the availability of numerous telepharmacy services in the UAE. In a Malaysian study, nearly all participants agreed with the idea that telepharmacy can help patients save money and travel time to reach healthcare facilities, while nearly half disagreed with the idea that telepharmacy services in Malaysia were available [19]. Similar results were also observed in an Indonesian study [13]. In Saudi Arabia, over two-thirds of the general population expressed confidence in telepharmacy's capacity to save time and energy, and they agreed with the notion of a wide array of telepharmacy services being available within the country [11].

Consistent with the findings reported in earlier research [14], the current study showed a positive association between participants' attitudes towards telepharmacy and their interest in using such services in the future. Furthermore, participants who had previously used telepharmacy services were more interested in using them in the future. The strong association between individuals' positive attitudes towards telepharmacy, their prior experience with telepharmacy, and their enthusiasm for future use can be explained by the convenience, accessibility, time-saving benefits, and numerous other advantages associated with these services. These elements collectively enhance the appeal of telepharmacy for those with a positive perspective and prior experience with this innovative service.

It is important to note that despite the opportunities telepharmacy offers, its implementation is not without challenges. Technical limitations, such as inadequate digital infrastructure, can impact service delivery, particularly in remote areas [20]. Additionally, legal and regulatory constraints may hinder the expansion of telepharmacy services, affecting patient access to these innovations. Security concerns related to data privacy also play a pivotal role, potentially limiting patient trust and willingness to utilize telepharmacy services [21].

### 4.1. Future Implications

The high interest in telepharmacy services underscores the need to integrate telepharmacy into the healthcare system and establish regulatory frameworks to ensure security, privacy, and reliability while expanding their accessibility to various populations. Furthermore, telepharmacy education should target both pharmacists and patients, as increased awareness can promote broader utilization. This strategy could be enhanced by implementing public awareness campaigns that educate the public about the benefits of telepharmacy, such as time and cost savings, improved clinical outcomes, enhanced adherence to treatment plans, and reduced patient visits and hospitalizations [22]. To fully benefit from telepharmacy, technological impediments must be overcome. Improving digital infrastructure, data security protocols, and legislative frameworks requires cooperation between technology companies, healthcare regulators, and policymakers to address these challenges. Future research should focus on customizing and developing telepharmacy models that overcome these obstacles and increase access to pharmacy services throughout the UAE.

### 4.2. Strengths and Limitations

The current study is not without its limitations. The use of convenience and snowball sampling may have introduced selection bias, as participants were not randomly selected, which might affect the generalizability of the results. The sampling methods employed were not derived from probability-based techniques, potentially limiting the ability to extrapolate these findings to the broader UAE population, as this approach is more susceptible to gathering data from a segment of the population that is not representative of the entire community. This particularly includes those who are more likely to engage in or have access to digital platforms and telepharmacy services.

Furthermore, the cross-sectional study design employed in this study only allowed for the observation of associations rather than cause-and-effect relationships. Moreover, the reliance on self-reported survey data could have rendered the study vulnerable to social desirability bias, as participants may have provided responses that they believed were socially acceptable rather than reflecting their true attitudes and behaviors.

It is noteworthy that the mean age of our sample (34 years) was relatively young. Considering the potential appeal of telepharmacy services to older populations, who may benefit significantly due to mobility issues or chronic health conditions, this age skew could limit the generalizability of our findings to the entire population. However, it is important to contextualize this within the demographic landscape of the UAE, where the median age is approximately 33 years [23], aligning closely with the mean age of our sample. Despite this, future studies should strive for a more varied age distribution to fully capture the perspectives of older individuals, who might represent a key demographic for telepharmacy services. Lastly, despite conducting face and content validity, other validation steps, including psychometric testing and construct validity, may have further improved the validity and reliability of the instrument. The key strength of this study lies in its significant contribution to the field, as it is the first of its kind in the UAE, providing valuable insights into people's attitudes and willingness to engage with telepharmacy services. In addition, the large sample size allowed for more robust conclusions to be drawn from the study.

## 5. Conclusions

In the current study, individuals demonstrated an interest and a positive attitude towards future telepharmacy utilization, which suggests a positive shift towards greater acceptance of this innovative approach compared to prior research. By expanding the accessibility of telepharmacy services and addressing the outlined technical, legal, and security challenges, the UAE can further harness individuals' interest in using telepharmacy services and promote positive outcomes for both patients and healthcare providers. However, future research that employs more robust, probability-based sampling techniques to validate these findings and ensure a comprehensive understanding of the public's attitudes towards telepharmacy is deemed necessary.

## Figures and Tables

**Table 1:** Sociodemographic characteristics of the study participants (*n* = 963).

	**Median (95% lower–upper CI) or frequency (%)**
Age	34 (33–36)
Sex	
Female	616 (64%)
Male	347 (36%)
Marital status	
Married	542 (56.3%)
Not married	421 (43.7%)
Education	
High school or less	198 (20.6%)
College	142 (14.7%)
University	623 (64.7%)
Occupation	
In the medical field	236 (24.5%)
In a nonmedical field	727 (75.5%)
Monthly income	
<10,000 AED	629 (65.3%)
10,000–30,000 AED	233 (24.2%)
>30,000 AED	101 (10.5%)
Having a chronic disease	
No	817 (84.8%)
Yes	146 (15.2%)
Using a chronic medication(s)	
No	17 (11.7%)
Yes	129 (88.3%)
Following a specific diet	
No	74 (50.8%)
Yes	72 (49.2%)
Do you or any member of your family suffer from chronic conditions?	
No	451 (46.8%)
Yes	512 (53.2%)
Insurance coverage	
No	185 (19.2%)
Yes	778 (80.8%)
Difficulties in accessing the pharmacy	
No	862 (89.5%)
Yes	101 (10.5%)
Have you ever used telepharmacy services before?	
No	834 (86.6%)
Yes	129 (13.4%)
Are you interested in using telepharmacy services in the future?	
No	200 (20.8%)
Yes	763 (79.2%)

Abbreviation: AED: the United Arab Emirates dirham.

**Table 2:** Public attitudes towards telepharmacy services (*n* = 963).

	**Strongly disagree (%)**	**Disagree (%)**	**Neutral (%)**	**Agree (%)**	**Strongly agree (%)**	**Corrected item–total correlation**	**Cronbach's alpha if item deleted**
I can use many telepharmacy services in the United Arab Emirates.	90 (9.3%)	197 (20.5%)	368 (38.2%)	274 (28.5%)	34 (3.5%)	0.38	0.90
I like using telepharmacy services.	64 (6.6%)	101 (10.5%)	263 (27.3%)	461 (47.9%)	74 (7.7%)	0.73	0.86
Telepharmacy services are important to be able to communicate with medical practitioners anytime and anywhere.	58 (6%)	77 (8%)	180 (18.7%)	525 (54.5%)	123 (12.8%)	0.75	0.86
Telepharmacy helps to save time and effort.	66 (6.9%)	62 (6.4%)	153 (15.9%)	537 (55.8%)	145 (15.1%)	0.77	0.86
Telepharmacy helps to reduce service costs.	67 (7%)	131 (13.6%)	233 (24.2%)	437 (45.4%)	95 (9.9%)	0.70	0.87
I am willing to pay for telepharmacy services.	91 (9.4%)	137 (14.2%)	274 (28.5%)	396 (41.1%)	65 (6.7%)	0.65	0.87
I would recommend telepharmacy services to my friends and family.	62 (6.4%)	87 (9%)	277 (28.8%)	450 (46.7%)	87 (9%)	0.79	0.86

**Table 3:** Factors associated with the willingness to utilize telepharmacy services.

	**AOR**	**95% confidence interval**		**Tolerance**	**VIF**
		**Lower**	**Upper**		
Age	0.993	0.977	1.010	0.580	1.724
Attitude score^∗∗^	1.147	1.112	1.182	0.988	1.012
Sex					
Male^a^	—	—	—	0.951	1.051
Female^b^	1.084	0.758	1.551		
Marital status					
Married^a^	—	—	—	0.610	1.639
Not married^b^	1.254	0.807	1.948		
Education					
High school or less^a^	—	—	—	0.878	1.140
College^b^	1.100	0.706	1.714		
University^b^	0.876	0.495	1.548		
Working status					
Medical field^a^	—	—	—	0.874	1.144
Nonmedical field^b^	0.896	0.580	1.385		
Income					
<10,000 AED^a^	—	—	—	0.895	1.117
10,000–30,000 AED^b^	0.890	0.501	1.580		
>30,000 AED^b^	1.458	0.933	2.277		
Insurance coverage					
No^a^	—	—	—	0.908	1.101
Yes^b^	1.028	0.658	1.606		
Difficulty to reach the pharmacy					
No^a^	—	—	—	0.981	1.019
Yes^b^	1.156	0.652	2.047		
Chronic disease					
No^a^	—	—	—	0.946	1.057
Yes^b^	1.080	0.764	1.526		
Have you ever used telepharmacy services before?^∗∗^					
No^a^	—	—	—	0.983	1.017
Yes^b^	3.270	1.692	6.320		

Abbreviation: VIF: variance inflation factor.

^a^Reference group.

^b^Test groups.

^**^ *p* value < 0.001.

## Data Availability

The quantitative data used to support the findings of this study are available from the corresponding author upon request.

## References

[1] Ryu S. (2012). Telemedicine: opportunities and developments in member states: report on the second global survey on eHealth 2009 (global observatory for eHealth series, volume 2). *Healthcare Informatics Research*.

[2] What is telepharmacy? eVisit. https://evisit.com/resources/what-is-telepharmacy.

[3] Kemp S. (2024). Digital 2024: the United Arab Emirates — Data reportal – global digital insights. https://datareportal.com/reports/digital-2024-united-arab-emirates.

[4] Alshamsi A. I. (2024). A review of the United Arab Emirates healthcare systems on medical tourism and accreditation. *Frontiers in Health Services*.

[5] Win A. Z. (2017). Telepharmacy: time to pick up the line. *Research in Social and Administrative Pharmacy*.

[6] Poudel A., Nissen L. (2016). Telepharmacy: a pharmacist&rsquo;s perspective on the clinical benefits and challenges. *Integrated Pharmacy Research and Practice*.

[7] Lam A. Y., Rose D. (2009). Telepharmacy services in an urban community health clinic system. *Journal of the American Pharmacists Association*.

[8] Clifton G. D., Byer H., Heaton K., Haberman D. J., Gill H. (2003). Provision of pharmacy services to underserved populations via remote dispensing and two-way videoconferencing. *American Journal of Health-System Pharmacy*.

[9] Casey M. M., Sorensen T. D., Elias W., Knudson A., Gregg W. (2010). Current practices and state regulations regarding telepharmacy in rural hospitals. *American Journal of Health-System Pharmacy*.

[10] Vision 2021 and health |the official portal of the UAE government. 2022. https://u.ae/en/about-the-uae/strategies-initiatives-and-awards/strategies-plans-and-visions/strategies-plans-and-visions-untill-2021/vision-2021-and-health.

[11] Alnajrani R. H., Alnajrani N. R., Aldakheel F. S. (2022). An assessment of the knowledge, perception, and willingness to use telepharmacy services among the general public in the Kingdom of Saudi Arabia. *Cureus*.

[12] Alanazi A., Albarrak A., Alanazi A., Muawad R. (2021). 5PSQ-184 Knowledge and attitude assessment of pharmacists toward telepharmacy in Riyadh City, Saudi Arabia. *Section 5: Patient safety and quality assurance*.

[13] Tjiptoatmadja N. N., Alfian S. D. (2022). Knowledge, perception, and willingness to use telepharmacy among the general population in Indonesia. *Frontiers in Public Health*.

[14] Dat T., Tran T. D., My N. T. (2022). Pharmacists’ perspectives on the use of telepharmacy in response to COVID-19 pandemic in Ho Chi Minh City, Vietnam. *Journal of Pharmacy Technology*.

[15] Hasan Ibrahim A. S., Barry H. E., Hughes C. M. (2023). GPs’ and pharmacists’ views of integrating pharmacists into general practices: a qualitative study. *British Journal of General Practice*.

[16] Abu-Farha R., Alzoubi K. H., Abu Assab M. (2023). Perception and willingness to use telepharmacy among the general population in Jordan. *Patient Preference and Adherence*.

[17] Aziz M. M., Masood I., Yousaf M., Saleem H., Ye D., Fang Y. (2018). Pattern of medication selling and self-medication practices: a study from Punjab, Pakistan. *PLoS One*.

[18] Mamo S., Ayele Y., Dechasa M. (2018). Self-medication practices among community of Harar City and its surroundings, Eastern Ethiopia. *Journal of Pharmaceutics*.

[19] Elnaem M. H., Akkawi M. E., Al-shami A. K., Elkalmi R. (2022). Telepharmacy knowledge, perceptions, and readiness among future Malaysian pharmacists amid the COVID-19 pandemic. *Indian Journal of Pharmaceutical Education and Research*.

[20] Nwachuya C. A., Umeh A. U., Ogwurumba J. C., Chinedu-Eze I. N., Azubuike C. C., Isah A. (2023). Effectiveness of telepharmacy in rural communities in Africa: a scoping review. *Journal of Pharmacy Technology*.

[21] Umar A. K., Limpikirati P., Zothantluanga J. H., Shumkova M. M., Prosvirkin G., Luckanagul J. A. (2024). Telepharmacy: a modern solution for expanding access to pharmacy services. *Artificial Intelligence, Big Data, Blockchain and 5G for the Digital Transformation of the Healthcare Industry*.

[22] Iftinan G. N., Elamin K. M., Rahayu S. A., Lestari K., Wathoni N. (2023). Application, benefits, and limitations of telepharmacy for patients with diabetes in the outpatient setting. *Journal of Multidisciplinary Healthcare*.

[23] United Arab Emirates’s median age Data demographics on world economics. https://www.worldeconomics.com/Demographics/Median-Age/United%2520Arab%2520Emirates.aspx.

